# MiR-24-BIM-Smac/DIABLO axis controls the sensitivity to doxorubicin treatment in osteosarcoma

**DOI:** 10.1038/srep34238

**Published:** 2016-09-29

**Authors:** Yangbai Sun, Nengbin He, Yang Dong, Chaoyin Jiang

**Affiliations:** 1 Department of Plastic and Reconstructive Surgery, Shanghai Ninth People’s Hospital, Shanghai Jiaotong University School of Medicine, Shanghai, China; 2 Department of Orthopedic Surgery, Shanghai Sixth People’s Hospital, Shanghai Jiaotong University, Shanghai, China

## Abstract

Emerging evidence shows that microRNAs (miRNAs) act as critical regulators in the progression and chemoresistance of multiple tumors, including osteosarcoma (OS). In this study, we found that the level of miR-24 was increased in OS patients’ serum, tumor tissues and OS cell lines. Furthermore, we found that knockdown of miR-24 by its specific inhibitors significantly increased the therapeutic effect of doxorubicin (DOX) on OS cell lines (MG-63 and HOS). Moreover, miR-24 inhibitors resensitized the doxorubicin-resistant MG-63 cells (MG-63/R) and HOS cells (HOS/R) to DOX. As the gene of Bcl-2 interacting mediator of cell death (BIM) was proved to be a target of miR-24 in MG-63/R cells, we further observed that the miR-24 inhibitors promoted the DOX-induced apoptosis via mitochondrial pathway. In addition, results of immunoprecipitation showed the release of second mitochondria derived activator of caspase/ direct IAP binding protein with low pI (Smac/DIABLO) abolished the biological activity of X-linked inhibitor of apoptosis protein (XIAP) by binding with it, which subsequently induced the activation of caspase 9, 7 and 3. In summary, those results strongly suggest that the miR-24-BIM-Smac/DIABLO axis might be a novel target for the treatment of OS.

Osteosarcoma (OS) is one of the most frequent primary malignant bone cancer affecting children and adolescents^1^. Though advances of modern treatments such as surgery, radiotherapy, and chemotherapy are improved, no substantial change in survival has been seen over the past 20 years, and the rate of long-term survival in patients with advanced OS remains very low^2^,^3^. Thus, understanding the mechanisms underlying OS as well as identifying new molecular targets is necessary to develop novel treatment strategies. Doxorubicin (DOX), an anthracycline antibiotic, is one of the most effective and widely used drugs in the treatment of multiple cancers, including OS^4^,^5^. However, clinical application of DOX is limited by harsh side effects such as dose dependent and cumulative cardiotoxicity that can lead to cardiac dysfunction^6^. Therefore, understanding the mechanisms underlying OS as well as reducing the dose of DOX and reversing the chemoresistance is of great importance.

Induction of mitochondrial apoptosis is an important mechanism for the anti-tumor effect of chemotherapeutic drugs. The pathway of mitochondrial apoptosis is initiated by the cellular stress (such as DOX-induced DNA damage) which induces the alterations of the outer mitochondrial membrane potential (MMP) and permeability. Subsequently, the apoptotic factors (such as Smac/DIABLO) will be released into the cytoplasm from the damaged mitochondria. As a result of this process, the caspases-dependent apoptosis finally occurs^7^,^8^.

MiRNAs represent a class of short noncoding RNA sequence that negatively regulate gene expression at the posttranscriptional level by binding to the 3′-untranslated regions of their target mRNAs^9^. Researches have demonstrated that miRNAs are involved in the regulation of a variety of biological processes including cell proliferation, differentiation, and apoptosis by regulating as much as 60% of the human protein coding genes^10^,^11^,^12^. In addition, miRNAs act as oncogenes or tumor suppressors depending on the role of their target genes^13^,^14^.

In the present study we examined the expression of miR-24 in a variety of OS cell lines and primary tumor samples from patients. We demonstrated that miR-24 controls OS cell sensitivity to DOX by targeting the BIM gene suggesting the miR-24-BIM-Smac/DIABLO axis to play a key role in OS cells sensitivity to DOX.

## Materials and Methods

### Ethics statement

The present study was conducted in accordance with the Declaration of Helsinki and the guidelines of the Ethics Committee of Shanghai Sixth People’s Hospital, Shanghai, China. The all experimental protocol was approved by Ethics Committee of Shanghai Sixth People’s Hospital, Shanghai, China. All patients and/or their parents gave their informed consent prior to inclusion in the study.

### Clinical specimens

Forty-five pairs of primary OS tissue samples and adjacent non-tumor tissue samples were obtained from patients who underwent tumor resection in Shanghai Sixth People’s Hospital from 1/2013 to 03/2015. Both tumor and noncancerous samples were confirmed histologically. In addition, 124 serum samples, including 62 OS patients and 62 healthy controls, were also collected from the same hospital. The use of clinical tissues and serum for this study was approved by the Hospital’s Protection of Human Subjects Committee. All patients and/or their parents gave their informed consent prior to inclusion in the study.

### Cell lines and cell culture

Osteosarcoma cell lines MG-63, HOS, Saos-2 and U-2 OS were originated from ATCC. The cells were maintained in DMEM supplemented with 10% FBS at 37 °C in a humidified atmosphere containing 5% CO2. To study the role of miR-24 in chemoresistance, we established DOX-resistant MG-63 (MG-63/R) and DOX-resistant HOS (HOS/R) cell lines by stepwise exposure of primary MG-63/HOS cells to increasing concentrations of DOX (Sigma-Aldrich, USA). The concentration of DOX was ranged from 0.1 μg/ml to 0.5 μg/ml and the MG-63/R cells were exposure to DOX over a time period of 12 months. To eliminate the influence of residual DOX in culture medium, the MG-63/R and HOS/R cells were cultured in DOX-free DMEM for two weeks before the following experiments were performed.

### Transfection

The OS cells were transfected with 50 pmol/ml miR-24 mimics (5′-UGGCUCAGUUCAGCAGGAACAG-3′), 50 pmol/ml negative control oligo-nucleotide (miR-NC, 5′-AGGUAGCAGCAGUCGCUGAUCA-3′), 50 pmol/ml 2′-Omethyl modified miR-24 inhibitors (anti-miR-24, 5′-CUGUUCCUGCUGAACUGAGCCA-3′) or 50 pmol/ml BIM siRNA (5′-GACCGAGAAGGUAGACAAUUU-3′) by using Lipofectamine 2000 (Invitrogen, USA) according to the manufacturer’s guidance. Oligo-nucleotides were purchased from Genepharma Company (China).

### RNA extraction and qRT-PCR

Total RNA from patients’ tissues, serum and cell lines were extracted using Trizol (Invitrogen, USA) according to the manufacturer’s instructions. For qRT-PCR analysis of BIM mRNA expression, the cDNA synthesis was carried out by the SuperScript II (Invitrogen) followed by the PCR performed by using TaqMan Gene Expression Assay (Applied Biosystem, Life Technologies Corporation, USA). GAPDH was used as an internal control. For analysis of miR-24 expression, mature miRNA was detected by using TaqMan microRNA assays that were specific for miR-24 (Applied Biosystem), and U6 snRNA was used as the internal control. The relative expression of BIM and miR-24 was determined using the 2^−△△CT^ analysis method^15^.

### Plasmid construction and luciferase reporter assay

The open reading frame of XIAP gene was amplified by PCR with the cDNA as template and cloned into the pcDNA3.1 vector (Life Technologies, USA), and the recombinant plasmid was named pcDNA3.1-XIAP. The 3′ UTR of BIM gene was amplified and cloned into the downstream of the Luciferase stop codon in the pMIR-REPORT™ miRNA Expression Reporter Vector (Life Technologies), giving rise to the pMIR-BIM 3′ UTR construct. The corresponding BIM mutant constructs were created by *in vitro* site-directed mutagenesis kit (Takara, Japan) based on the wild-type recombinant reporter vector. The constructs were confirmed by bidirectional sequencing. To perform the luciferase reporter assay, MG-63/R and HOS/R cells were cultured in the 24-well plates, and each was cotransfected with 50 pmol/ml miR-24 mimics (or miR-NC) plus 2 μg/ml luciferase reporter constructs and 100 ng/ml Renilla luciferase pRL-TK vector (Promega, USA). Forty-eight hours after transfection, luciferase activities were measured by using the Dual-Luciferase Reporter Kit (Promega) according to the manufacturer’s instructions. Firefly luciferase activity was normalized to the Renilla luciferase activity.

### Mitochondria isolation

MG-63/R and HOS/R cells were harvested by centrifugation at 500 × g for 10 min at 4 °C. The cells were washed once with ice-cold PBS and resuspended in digitonin lysis buffer (150 μg Digitonin dissolved in 1 ml 500 mM sucrose solutions) followed by centrifugation at 1,200 × g for 5 min at 4 °C. The supernatant was then centrifuged again at 12,000 × g for 30 min at 4 °C. The resulting supernatant was collected as cytoplasmic fraction, and the remaining organelles were considered as the mitochondrial fraction.

### Immunoprecipitation

The cytoplasmic fraction was collected as described above, and then the lysates were incubated with primary antibody of Smac/DIABLO (Cell Signaling Technologies, USA) overnight at 4 °C followed by 2 h of incubation of protein A agarose beads. After washing the beads with cold digitonin lysis buffer, proteins were removed from the beads by boiling in sodium dodecyl sulfate (SDS) sample buffer and analyzed by western blotting analysis.

### Western blot analysis

Protein concentrations were determined by BCA protein assay kit (Thermo Scientific, USA). The protein samples with equal quality were separated by SDS-PAGE and transferred to a PVDF membrane (Millipore, USA). Membranes were blocked with 5% skim milk for 1 h at room temperature and probed with primary antibodies overnight (including anti-human BIM, Smac/DIABLO, XIAP, caspase-9, caspase-7, caspase-3 and β-actin. All of the antibodies were purchased from Cell Signaling Technologies) followed by incubation with a HRP-conjugated secondary antibody for 2 h. The blots were detected with an enhanced chemilu-minescence detection kit (Pierce, USA).

### Cell viability

After the tumor cells were treated with Oligo-nucleotides and DOX, the cell viability was measured by MTT assay as previously described^16^.

### Apoptosis and mitochondrial membrane potential (MMP, ∆Ψ_m_) analysis

The MG-63/R and HOS/R cells in six-well plates were treated with RNAs and DOX, alone or in combination. The cells were harvested and stained with propidium iodide and FITC-annexin V according to the manufacturer’s instructions (Sigma-Aldrich, USA). The stained cells were analyzed by flow cytometry (Becton Dickinson, USA). For mitochondrial membrane potential (MMP, ∆Ψ_m_) detection, 5,5′,6,6′-Tetrachloro-1,1′,3,3′-tetraethyl imidacarbo cyanine iodide (JC-1, Molecular Probes, USA) was used as an indicator^17^. Cells were collected and washed with ice-cold PBS twice, and then stained with JC-1 at a concentration of 5 μM. Following a 20 min incubation period at 37 °C in the dark, ∆Ψ_m_ was determined by flow cytometry analysis.

### Statistical analysis

Data from studies were expressed as the mean ± standard deviation. The statistical significances of differences between groups were determined using Student’s t test performed by SPSS 14.0 software. Difference was considered statistically significant if p < 0.05. Data from this paper is supported by at least three independent experiments.

## Results

### MiR-24 is up-regulated in OS patients’ serum and tumor cells *in vitro* and vivo

**Figure 1:**
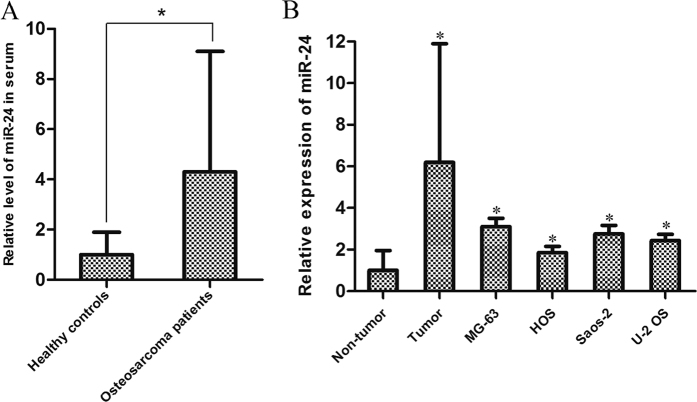
MiR-24 is expressed in tissues and serum from patients in addition to OS cell lines *in vivo*. (**A**) The expression of miR-24 in the serum of 62 OS patients and 62 healthy controls was detected by using qRT-PCR analysis. *p < 0.05. The means of RNA expression were determined by using the 2^−△△CT^ analysis method, and normalized to U6 snRNA. The average miR-24 level in the serum of all healthy controls was normalized to the average serum level of all OS patients and a *p* < 0.05 was considered significant. (**B**) qRT-PCR analysis of miR-24 expression was conducted on 45 pairs of primary OS tissue samples and adjacent non-tumor tissue samples as well as four OS cell lines. *p < 0.05 *vs.* non-tumor tissues group. The average miR-24 levels of all the four OS cell lines and all OS tumor tissues were normalized to the average level of all non-tumor tissues and a *p* < 0.05 was considered significant. All data represent the mean ± SD from at least three independent experiments in each condition.

RT-qPCR was used to see if the expression level of miR-24 was elevated in OS patients’ serum. As shown in Fig. 1A, the miR-24 levels were significantly up-regulated in OS patients’ serum compared with the healthy controls’ (the expression of miR-24 increased 4.31 fold in OS patients’ serum *vs.* healthy controls’, *P* < 0.05). Next, we detected the miR-24 expression levels between OS tissues and the paired non-tumor tissues. As shown in Fig. 1B, compared with non-tumor tissues, the expression of miR-24 was significantly increased in OS tissues. In addition, an increased miR-24 expression level was also observed in MG-63, HOS, Saos-2 and U-2 OS cell lines compared with the cancer-free tissues *in vitro* (Fig. 1B). These data suggest that miR-24 plays potential role in OS development.

### MiR-24 determines the sensitivity of OS cells to doxorubicin

**Figure 2:**
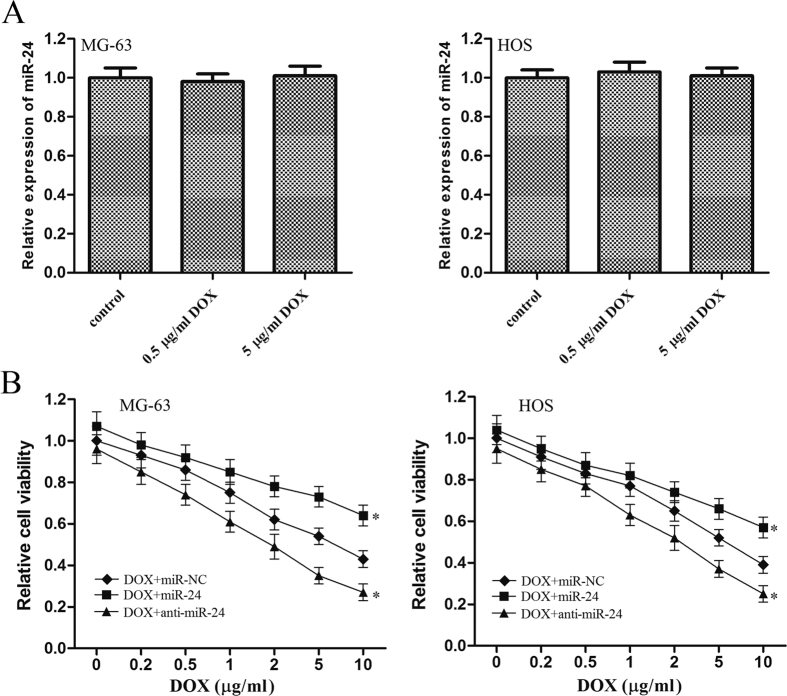
MiR-24 is associated with the chemotherapy in OS. (**A**) 48 h after treatment with DOX (0.5 μg/ml or 5 μg/ml), the expression of miR-24 was detected by using qRT-PCR in MG-63 and HOS cells. Relative expression of miR-24 was normalized to the control cells. (**B**) MG-63 and HOS cells were transfected with miR-24 mimics or the inhibitors. 48 h after transfection, the cells were treated with DOX for another 48 h, MTT assay was then performed to evaluate the viability of OS cells. *p < 0.05 *vs.* DOX plus miR-NC group. All the data from the DOX + miR-24 group and DOX + anti-miR-24 group were normalized to the data of the miR-NC group and a *p* < 0.05 was considered significant. All data represent the mean ± SD from at least three independent experiments in each condition.

As the expression of miR-24 was not changed by the DOX treatment in OS cells (Fig. 2A), we observed that knockdown of miR-24 significantly increased the sensitivity of OS cells to DOX compared with the miR-NC group (Fig. 2B). Meanwhile, the anti-tumor effect of DOX was significantly impaired when the miR-24 but not the miR-NC was overexpressed (Fig. 2C). Therefore, we demonstrated that MiR-24 determines the sensitivity of OS cells to DOX.

### Knockdown of miR-24 reverses the doxorubicin-resistance in OS cells

**Figure 3:**
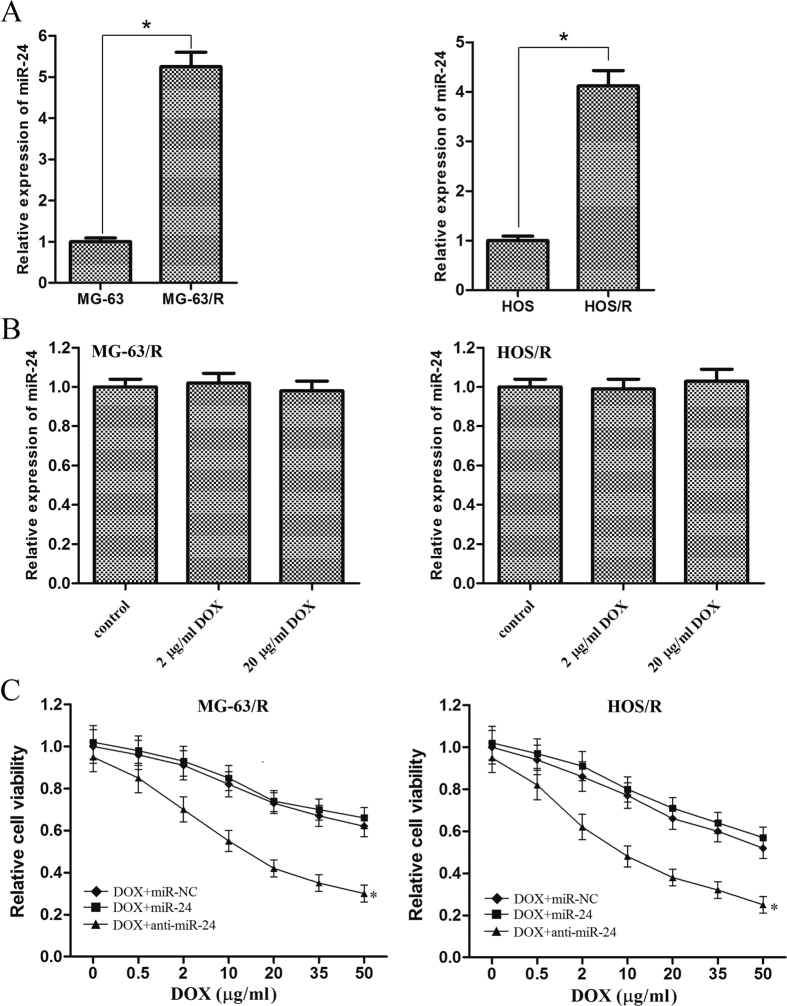
High expression level of miR-24 is associated with the DOX-resistance in MG-63 and HOS cells. (**A**) qRT-PCR analysis of miR-24 expression was conducted on MG-63, MG-63/R, HOS, HOS/R cells. *p < 0.05. The miR-24 expression levels of MG-63/R and HOS/R cells were normalized to MG-63 and HOS, respectively. A *p* < 0.05 was considered significant. (**B**) 48 h after treatment with DOX (2 μg/ml or 20 μg/ml), the expression of miR-24 was detected by using qRT-PCR in MG-63/R and HOS/R cells. The data from the treated groups were normalized to the data of the miR-NC group. (**C**) MG-63/R and HOS/R cells were transfected with 50 pmol/ml miR-NC, miR-24 mimics or anti-oligonucleotides. 48 hours after transfection, the cells were treated with DOX for another 48 h, MTT assay was then performed to evaluate the cell viability. *p < 0.05 *vs.* DOX plus miR-NC group. All the data from the DOX + anti-miR-24 group were normalized to the data of the miR-NC group and a *p* < 0.05 was considered significant. All data represent the mean ± SD from at least three independent experiments in each condition.

To explore the biological roles of miR-24 in the therapeutic effect of DOX on OS cells, we established DOX-resistant MG-63 (MG-63/R) and DOX-resistant HOS (HOS/R) cell lines by stepwise exposure to DOX over 12 month. As shown in Fig. 3A, obvious up-regulation of miR-24 was found in MG-63/R and HOS/R cells compared with their parental cells, respectively (the expression of miR-24 increased 5.25 fold in MG-63/R *vs.* MG-63, *P* < 0.05; the expression of miR-24 increased 4.12 fold in HOS/R *vs.* HOS, *P* < 0.05). Furthermore, as the expression of miR-24 was not changed by the DOX treatment in MG-63/R and HOS/R cells (Fig. 3B), we observed knockdown of miR-24 by its anti-oligonucleotides resensitized them to DOX-induced cell death (Fig. 3C). Interestingly, further up-regulation of miR-24 by the mimics in MG-63/R and HOS/R cells failed to protect them from the cytotoxicity of high concentration of DOX (Fig. 3C).

### BIM is the target of miR-24 in MG-63/R and HOS/R OS cells

**Figure 4:**
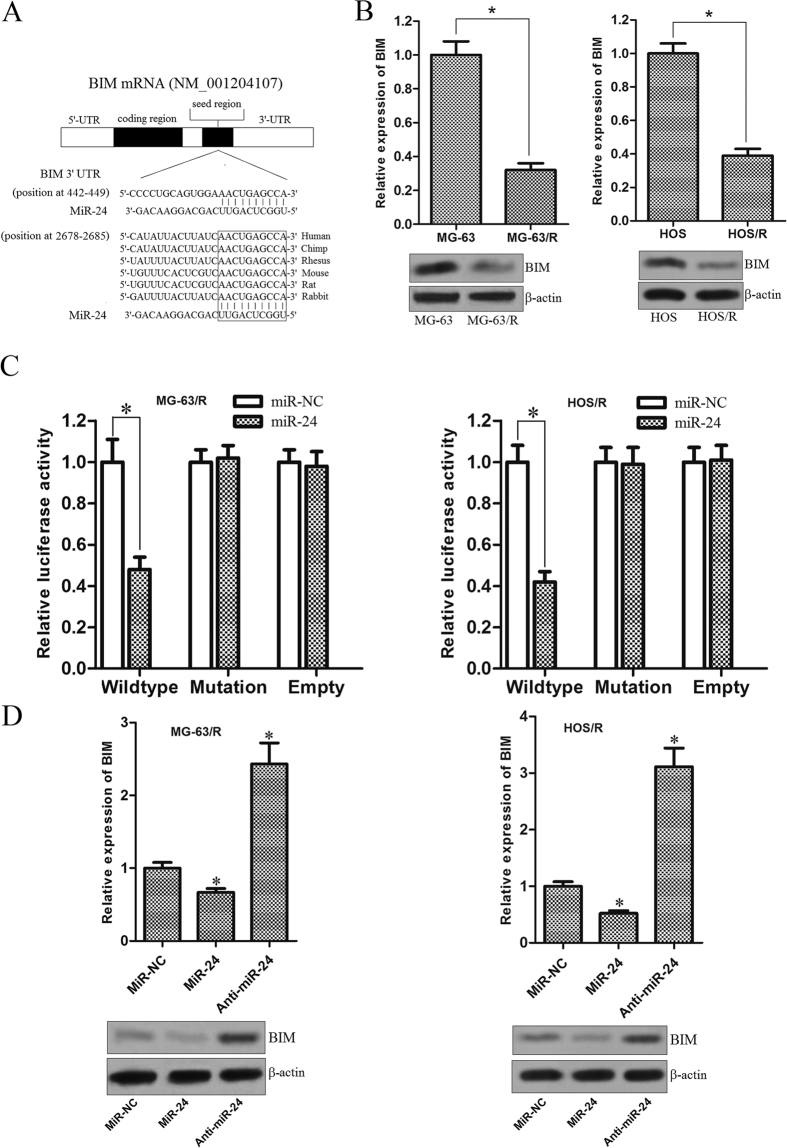
BIM is the target of miR-24 in DOX-resistant OS cells. (**A**) Putative miR-24 binding sequence in the 3′ UTR of BIM mRNA according to the three miRNA databases (TargetScan, miRanda, and PicTar). (**B**) The expression of BIM at mRNA level and protein level in MG-63, MG-63/R, HOS, and HOS/R cells was examined by RT-qPCR and western blot analysis, respectively. The mean of mRNA expression was determined by using the 2^−△△CT^ analysis method, and normalized to GAPDH RNA. *p < 0.05. The BIM expression levels of MG-63/R and HOS/R were normalized to MG-63 and HOS, respectively. A *p* < 0.05 was considered significant. **(C)** MG-63/R and HOS/R cells were transfected with pMIR reporters containing wild-type 3′-UTR of BIM or the mutant one, and miR-24 mimics or miR-NC were together introduced using Lipofectamine 2000 reagent. 48 h post transfection, the Firefly luciferase activity was measured, and normalized to the Renilla luciferase using the Dual-Luciferase Reporter System. *p < 0.05. The data from the co-transfection with miR-24 and wildtype reporter group were normalized to the data of the co-transfection with miR-NC and wildtype reporter group and a *p* < 0.05 was considered significant. (**D**) MG-63/R and HOS/R cells were transfected with 50 pmol/ml miR-NC, miR-24 mimics or anti-miR-24. After 72 hours incubation, RT-qPCR and western blot analysis were performed to evaluate the expression of BIM. *p < 0.05 *vs.* miR-NC group. The data from the miR-24 group and anti-miR-24 group were normalized to the data of the miR-NC group and a *p* < 0.05 was considered significant. All data represent the mean ± SD from at least three independent experiments in each condition.

It is well recognized that miRNAs play their functions by regulating the expression of target genes. To investigate the molecular mechanisms responsible for resensitizing the MG-63/R and HOS/R cells to DOX, three miRNA databases (TargetScan, miRanda, and PicTar) were used to predict miR-24 binding sites in human mRNA transcripts. We found BIM was commonly predicted by all of these predictive tools. Moreover, analysis of interspecies variation demonstrated that the miR-24 binding site in the 3′ UTR sequence of BIM is evolutionary conserved (Fig. 4A). Therefore, we inferred that the gene of BIM which is the pro-apoptotic member of Bcl-2 family proteins^18^ may be the key target correlating with the chemoresistance. In addition, we also found the expression level of BIM was significantly lower in MG-63/R and HOS/R cells compared with their parental cells, respectively (Fig. 4B). Taken together, we inferred that the down-regulation of BIM is caused by the overexpression of miR-24 in DOX-resistant OS cells. To validate if BIM is an actual target of miR-24 in MG-63/R and HOS/R, the wild-type and mutant 3′ UTR with the seed region of BIM was cloned downstream of the firefly luciferase gene in the pMIR reporters. As shown in Fig. 4C, a significant decrease of luciferase activity was observed specifically in wild-type 3′ UTR group (the activity of luciferase in miR-24 group decreased 52% compared to the miR-NC group in the MG-63/R cells, *P* < 0.05; the activity of luciferase in the miR-24 group decreased 58% compared to the miR-NC group in the HOS/R cells, *P* < 0.05), while in mutant 3′ UTR and empty control groups, this activity was abrogated when the MG-63/R and HOS/R cells were cotransfected with miR-24 mimics and pMIR reporters. Next, to confirm that miR-24 regulates the expression of BIM, the level of BIM mRNA and protein were measured after the miR-24 mimics or antagonist was transfected into MG-63/R and HOS/R cells. We observed that both BIM mRNA and protein expression were decreased after the miR-24 mimics transfection. In contrast, upon abrogation of miR-24 expression in MG-63/R and HOS/R by the anti-oligonucleotides, the expression of BIM was significantly up-regulated (Fig. 4D). Taken together, these results suggest that the BIM gene is a functional target of miR-24, may play an essential role in doxorubicin-resistant OS cells response to chemotherapy.

### Knockdown of miR-24 resensitizes MG-63/R and HOS/R cells to DOX-induced apoptosis via the mitochondrial pathway

**Figure 5:**
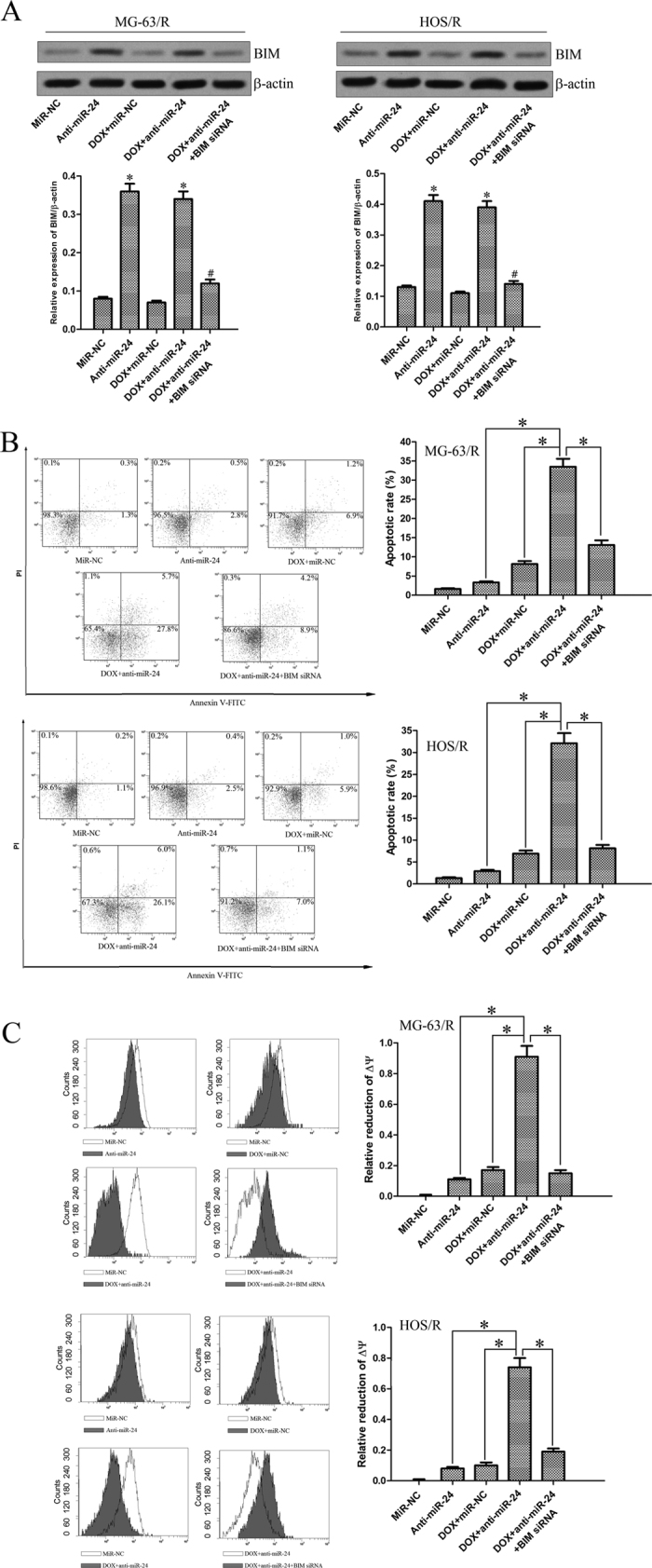
Co-treatment with anti-miR-24 and DOX triggered the mitochondrial apoptosis. (**A**) Western blot analysis of BIM protein expression in MG-63/R and HOS/R cells following treatment with DOX, anti-miR-24, and BIM siRNA. *p < 0.05 *vs.* miR-NC group. The data from the anti-miR-24 group and DOX + anti-miR-24 group were normalized to the data of the miR-NC group and a *p* < 0.05 was considered significant. ^#^*p* < 0.05 *vs.* DOX + anti-miR-24 group. The data from the DOX + anti-miR-24 + BIM siRNA group were normalized to the data of the DOX + anti-miR-24 group and a *p* < 0.05 was considered significant. (**B**) MG-63/R and HOS/R cells were transfected with 50 pmol/ml anti-miR-24 and 50 pmol/ml BIM siRNA for 48 h. Then, the cells were treated with 10 μg/ml DOX for incubation of another 48 h. The apoptotic rate was analyzed using Annexin V staining on flow cytometry. *p < 0.05. The data from the anti-miR-24 group, DOX + miR-NC group and DOX + anti-miR-24 + BIM siRNA group were normalized to the data of the DOX + anti-miR-24 group and a *p* < 0.05 was considered significant. (**C**) The mitochondrial membrane potential (∆Ψ_m_) was detected using JC-1 staining on flow cytometry. *p < 0.05. The data from the anti-miR-24 group, DOX + miR-NC group and DOX + anti-miR-24 + BIM siRNA group were normalized to the data of the DOX + anti-miR-24 group and a *p* < 0.05 was considered significant. All data represent the mean ± SD from at least three independent experiments in each condition.

To assess whether BIM regulates the sensitivity of MG-63/R and HOS/R cells to DOX, we inhibited the BIM expression by using siRNA. As shown in Fig. 5A, transfection of BIM siRNA significantly decreased the BIM protein level, even in the case of anti-miR-24 transfection (in MG-63/R cells, the expression of BIM decreased 64.7% in the DOX + anti-miR-24 + BIM siRNA group *vs.* DOX + anti-miR-24 group, *P* < 0.05; in HOS/R cells, the expression of BIM decreased 64.1% in the DOX + anti-miR-24 + BIM siRNA group *vs.* DOX + anti-miR-24 group, *P* < 0.05. Densitometry values were evaluated by Image J software). To analyze whether the anti-tumor efficacy caused by the co-treatment with miR-24 inhibitors and DOX was the result of cell apoptosis, Annexin V staining was performed. As shown in Fig. 5B, the percentage of apoptotic cells was markedly higher in the combination group with DOX and anti-miR-24 than in the anti-miR-24 group as well as DOX group (33.5% vs. 3.3% and 8.1% in MG-63/R cells, respectively, *P* < 0.05; 32.1% vs. 2.9% and 6.9% in HOS/R cells, respectively, *P* < 0.05). However, the apoptotic rate of cells co-treated with DOX, anti-miR-24 and BIM siRNA was lower than that of DOX plus anti-miR-24 co-treated cells (13.1% vs. 33.5% in MG-63/R cells, respectively, *P* < 0.05; 8.1% vs. 32.1% in HOS/R cells, respectively, *P* < 0.05). To further test whether combination triggered apoptosis is dependent on the mitochondrial pathway, the mitochondrial membrane potential (MMP, ∆Ψ_m_) was detected. We observed that the combination group with DOX and anti-miR-24 showed higher reduction of MMP than that of anti-miR-24 group as well as DOX group (0.91 vs. 0.11 and 0.17 in MG-63/R cells, respectively, *P* < 0.05; 0.74 vs. 0.08 and 0.10 in HOS/R cells, respectively, *P* < 0.05). As expected, the relative reduction of MMP was obviously lower in the treatment group with DOX, anti-miR-24 and BIM siRNA than in the DOX plus anti-miR-24 group (0.15 vs. 0.91 in MG-63/R cells, respectively, *P* < 0.05; 0.19 vs. 0.74 in HOS/R cells, respectively, *P* < 0.05) (Fig. 5C). Taken together, these results suggest that knockdown of miR-24 promotes the DOX-induced apoptosis and mitochondrial dysfunction by up-regulating the expression of BIM in DOX-resistant OS cells.

### Knockdown of miR-24 resensitizes MG-63/R and HOS/R cells to DOX through MiR-24-BIM-Smac/DIABLO axis

**Figure 6:**
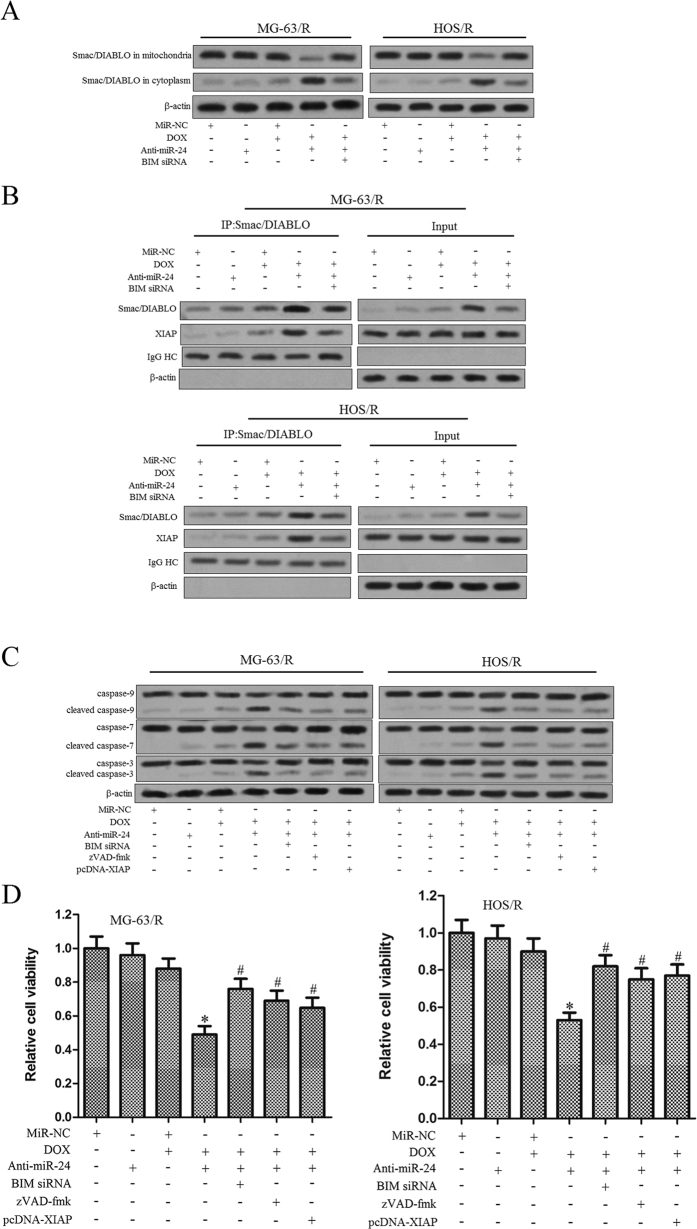
The role of miR-24-BIM-Smac/DIABLO axis is implicated in DOX-based treatment to MG-63/R and HOS/R OS cells response to DOX. (**A**) MG-63/R and HOS/R cells were transfected with 50 pmol/ml anti-miR-24 and 50 pmol/ml BIM siRNA for 48 h. Then, the cells were incubated for 48 hours with 10 μg/ml of DOX. After treatment, the mitochondria fraction and cytoplasm were separated, and the level of Smac/DIABLO in mitochondria or cytoplasm was evaluated by western blot analysis. (**B**) After the treatment as above, the interaction of Smac/DIABLO and XIAP was determined by co-immunoprecipitation of Smac/DIABLO. (**C**) Pre-treated with zVAD-fmk (10 μM), BIM siRNA or pcDNA-XIAP (2 μg/ml) significantly inhibited the cleavage of caspases when the MG-63/R and HOS/R cells were co-treated with anti-miR-24 and DOX. (**D**) After the treatment as above, the cell viability was determined by MTT assay. *p < 0.05 *vs.* DOX plus miR-NC group. The data from the DOX + anti-miR-24 group were normalized to the data of the DOX + miR-NC group and a *p* < 0.05 was considered significant. ^#^*p* < 0.05 *vs.* DOX plus anti-miR-24 group. The data from the DOX + anti-miR-24 + BIM siRNA group, DOX + anti-miR-24 + zVAD-fmk group and DOX + anti-miR-24 + pcDNA-XIAP group were normalized to the data of the DOX + anti-miR-24 group and a *p* < 0.05 was considered significant. All data represent the mean ± SD from at least three independent experiments in each condition.

Since our results demonstrated that knockdown of miR-24 in MG-63/R and HOS/R cells significantly up-regulated the expression level of BIM which is a pro-apoptotic protein located in the outer mitochondrial membrane^19^, we further investigated implications of the downstream components of the signal transduction pathway for apoptosis. As shown in Fig. 6A, we observed the Smac/DIABLO which is the mitochondria-derived apoptogenic protein, was released into cytoplasm from the mitochondria in the MG-63/R and HOS/R cells co-treated with miR-24 and DOX. Previous studies have demonstrated that one important biological function of Smac/DIABLO in cytoplasm is binding to X-linked inhibitor of apoptosis (XIAP), neutralizing its inhibitory effect on caspases and triggering intrinsic cell death^20^. Given the release of Smac/DIABLO in MG-63/R and HOS/R cells, we investigated the effects of the combination treatment on the interaction between Smac/DIABLO and XIAP. According to the results of immunoprecipitation, DOX alone treatment had little effect on the Smac/DIABLO and XIAP interaction, while the anti-miR-24 was shown to robustly enhance this interaction. As demonstrated by the IP assay where the effect of BIM siRNA was evaluated, transfection of BIM siRNA disrupted the release of Smac/DIABLO and the interaction between Smac/DIABLO and XIAP (Fig. 6B). These results suggest that the anti-miR-24 promotes the DOX-induced mitochondrial dysfuction by up-regulating the expression of BIM followed by the release of Smac/DIABLO from mitochondria and the subsequent neutralization of XIAP. Inhibition of XIAP triggers intrinsic apoptosis by activating the caspase-9, caspase-7 and caspase-3, we then investigated the effects of combination treatment DOX + anti-miR-24 on the caspase activation. As shown in Fig. 6C, we showed that combination treatment with DOX plus anti-miR-24 resulted in obvious cleavage of caspase-9, caspase-7 and caspase-3, and the activation of caspases could be impaired by the zVAD-fmk, BIM siRNA or the enforced expression of XIAP. These results suggest miR-24 + DOX-induced apoptosis and caspase activation is dependent on the BIM-Smac/DIABLO axis. Furthermore, as expected, both the BIM siRNA and pcDNA-XIAP significantly inhibited the cell death induced by the combination with DOX and anti-miR-24 in MG-63/R and HOS/R cells (Fig. 6D). Taken together, our data strongly suggested the important role of miR-24-BIM-Smac/DIABLO axis in sensitizing MG-63 and HOS OS cells to DOX.

## Discussion

Studies have demonstrated that miR-24 acts as an oncogene in multiple cancers. Du *et al*. demonstrated that miR-24 was up-regulated in breast cancer cells, enhancing tumor invasion and metastasis by targeting PTPN9 and PTPRF to promote the EGF signaling^21^. Le *et al*. also found that the expression level of miR-24 in the serum of patients with lung cancer was increased as compared to the serum of normal controls^22^. In hepatocellular carcinoma, overexpression of miR-24 was associated with increase cell proliferation, migration and invasion through targeting sex-determining region Y (SRY)-box 7 (SOX7)^23^. However, expression of miR-24 in OS and its detailed molecular function has not been further investigated. In this paper, we found a significant increase of miR-24 expression in the serum of patients with OS as compared to the control non-tumor patient (*P* < 0.05). The up-regulation of miR-24 was also detected in OS tissues and cell lines compared with the tumor-free tissues (*P* < 0.05). These results suggest that the overexpression of miR-24 may be essential for the development of OS.

Chemoresistance is a major challenge to effective cancer treatment, and efforts to increase the curative effect of chemotherapeutics have provided limited results. MiRNAs have been demonstrated to regulate multiple tumor-related genes, some of them implicated in tumor resistance to chemotherapy. Such examples include work by Xie X *et al*. who identified a novel regulatory pathway for apoptosis and doxorubicin-resistance involving miR-125b and its target Mcl-1 as a potential target for reversing the DOX chemoresistance in breast cancer^24^. High expression levels of miR-27a/b detected in serum correlated with poor response to chemotherapy in patients with esophageal cancer^25^. MiR-193b was found to be downregulated in hepatocellular carcinoma. Enforced expression of miR-193b significantly enhanced the curative effect of cisplatin^26^. Combination treatment with tamoxifen and anti-miR-181b or anti-miR-221 proved to reduce breast tumor size^27^.

In our present studies, we found that inhibition of miR-24 increased the cytotoxicity effect of DOX against OS cell lines. Furthermore, we found the expression of miR-24 to be significantly up-regulated in DOX-resistant MG-63 and HOS cells. Additional increase in the level of miR-24 by its mimics didn’t influence the anti-tumor effect of DOX in MG-63/R and HOS/R cells. However, knocking down miR-24 in both cells lines resulted in increased sensitivity to DOX.

We further identified BIM as the target of miR-24 in OS. BIM belongs to the BH3-only subfamily of Bcl-2 family proteins^28^. Studies have demonstrated that BIM plays a pro-apoptotic role by triggering Bak and Bax, which are additional apoptosis stimuli^29^,^30^. Activation of Bak and Bax leads to homo-oligomerization and assembly within the mitochondrial outer membrane followed by formation of the mitochondrial membrane permeability transition pore (PTP) with subsequent release of apoptotic factors (e.g., Smac/DIABLO), caspase activation and cell death^31^,^32^,^33^. It is well known that up-regulation of BIM in the outer mitochondrial membrane results in the mitochondrial dysfunction characterized by alteration of the mitochondrial membrane potential (∆Ψ_m_) and initiation of the intrinsic apoptotic pathway^34^. BIM is frequently downregulated in human cancers. BIM downregulation promotes cancer development and is associated with poor response to targeted therapy. Tumor cells with low levels of BIM could evade apoptosis and survive in the crucial environment even when surrounded by anti-tumor drugs^35^,^36^.

**Figure 7:**
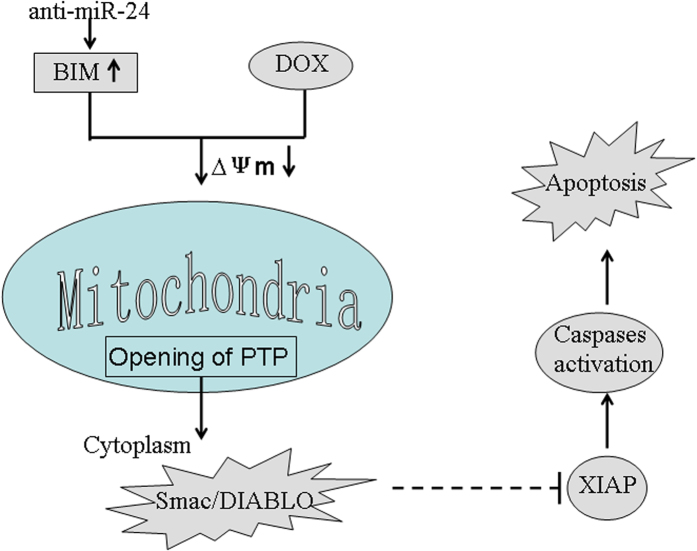
Schema of the predicted mechanisms implicated in OS cells response to DOX. Anti-miR-24 promotes the DOX-induced mitochondrial dysfuction, as determined by a decrease in MMP by up-regulating the expression of BIM. As a result, the opening of PTP is induced, followed by the release of releasing Smac/DIABLO from the mitochondria into the cytoplasm. Cytoplasmic Smac/DIABLO then binds to XIAP and to inactivates it, leading to the effector caspases activation and the final occurrence of and apoptosis.

BIM was downregulated in the DOX resistant MG-63 and HOS cells where miR-24 was upregulated. Further inhibition of miR-24 and treatment of these cells with DOX resulted in DOX-induced BIM upregulation, mitochondrial damage and cell death. We further demonstrate that translocation of Smac/DIABLO and binding to XIAP, an anti-apoptotic molecule, was the mechanism implicated in OS cells response to the combination treatment with anti-miR-24 and DOX. Binding to XIAP results in decrease in XIAP-mediated caspase inhibition and apoptotic cell death^37^,^38^,^39^. Further inhibition of XIAP using pcDNA-XIAP transfection, abolished caspases activation when the cells were subjected to combination treatment with anti-miR-24 and DOX. In summary, we demonstrate that knockdown of miR-24 promotes the DOX-induced mitochondrial dysfuction by up-regulating the expression of BIM. As a result, the opening of PTP is induced, followed by the release of Smac/DIABLO from the mitochondria into the cytoplasm. The cytoplasmic Smac/DIABLO then binds to XIAP to inactivate it, leading to the activation of caspase 9, 7 and 3 and apoptosis (Fig. 7).

In conclusion, here we provide a novel therapeutic strategy to enhance OS cells response to DOX, one of the chemotherapeutic agents used as part of the standard regimen to treat OS. However, further studies are necessary to develop suitable means to inhibit miRNA expression to enhance DOX efficacy in OS.

## Additional Information

**How to cite this article**: Sun, Y. *et al*. MiR-24-BIM-Smac/DIABLO axis controls the sensitivity to doxorubicin treatment in osteosarcoma. *Sci. Rep.* **6**, 34238; doi: 10.1038/srep34238 (2016).
